# Change in pulse pressure and cardiovascular outcomes after percutaneous coronary intervention: The CLIDAS study

**DOI:** 10.1016/j.ijcha.2024.101430

**Published:** 2024-05-24

**Authors:** Kotaro Nochioka, Masaharu Nakayama, Naoyuki Akashi, Tetsuya Matoba, Takahide Kohro, Yusuke Oba, Tomoyuki Kabutoya, Yasushi Imai, Kazuomi Kario, Arihiro Kiyosue, Yoshiko Mizuno, Takamasa Iwai, Yoshihiro Miyamoto, Masanobu Ishii, Taishi Nakamura, Kenichi Tsujita, Hisahiko Sato, Hideo Fujita, Ryozo Nagai, on behalf of the CLIDAS research group

**Affiliations:** a Division of Cardiovascular Medicine, Tohoku University Hospital, Seiryo-machi 1-1, Aoba-ku, Sendai 981-0933, Japan; b Department of Medical Informatics, Tohoku University Graduate School of Medicine, 1-1-1 Seiryo-machi, Aoba-ku, Sendai 981-0933, Japan; c Division of Cardiovascular Medicine, Jichi Medical University Saitama Medical Center, 1-847 Amanuma-cho, Omiya-ku, Saitama 330-8503, Japan; d Department of Cardiovascular Medicine, Kyushu University Graduate School of Medical Sciences, 3-1-1 Maidashi, Higashi-ku, Fukuoka 812-8582, Japan; e Department of Clinical Informatics, Jichi Medical University School of Medicine, 3311-1 Yakushiji, Shimotsuke, Tochigi 329-0498, Japan; f Division of Cardiovascular Medicine, Department of Medicine, Jichi Medical University School of Medicine, 3311-1 Yakushiji, Shimotsuke, Tochigi 329-0498, Japan; g Division of Clinical Pharmacology, Department of Pharmacology, Jichi Medical University, 3311-1 Yakushiji, Shimotsuke, Tochigi 329-0498, Japan; h Department of Cardiovascular Medicine, University of Tokyo Hospital, 7-3-1 Hongo, Bunkyo-ku, Tokyo 113-8655, Japan; i Development Bank of Japan Inc., 1-9-6 Otemachi, Chiyoda-ku, Tokyo 100-8178, Japan; j Department of Cardiovascular Medicine, National Cerebral and Cardiovascular Center, 6-1 Kishibe-Shimmachi, Suita 564-8565, Japan; k Open Innovation Center, National Cerebral and Cardiovascular Center, 6-1 Kishibe-Shimmachi, Suita 564-8565, Japan; l Department of Cardiovascular Medicine, Graduate School of Medical Sciences, Kumamoto University, 1-1-1 Honjo, Chuo-ku, Kumamoto 860-8556, Japan; m Department of Cardiovascular Medicine, Department of Medical Information Science, Graduate School of Medical Sciences, Kumamoto University, 1-1-1 Honjo, Chuo-ku, Kumamoto 860-8556, Japan; n Precision Inc., 4-2-5 Hongo, Bunkyo-ku, Tokyo 113-0033, Japan; o Jichi Medical University School of Medicine, 3311-1 Yakushiji, Shimotsuke, Tochigi 329-0498, Japan

**Keywords:** Pulse pressure, Coronary artery disease, Prognosis

## Abstract

**Background:**

Limited data exist on the prognostic value of changes in pulse pressure (PP, the difference between systolic and diastolic blood pressure) during hospitalization for patients with coronary artery disease who have undergone percutaneous coronary intervention (PCI).

**Methods:**

In the Clinical Deep Data Accumulation System (CLIDAS), we studied 8,708 patients who underwent PCI. We aimed to examine the association between discharge PP and cardiovascular outcomes. PP was measured before PCI and at discharge. Patients were divided into five groups (quintiles) based on the change in PPQ1 (−18.0 ± 9.9 mmHg), Q2 (−3.8 ± 2.6), Q3 (reference; 3.7 ± 2.0), Q4 (11.3 ± 2.6), and Q5 (27.5 ± 11.2). We then analyzed the relationship between PP change and outcomes.

**Results:**

The mean patient age was 70 ± 11 years, with 6,851 (78 %) men and 3,786 (43 %) having acute coronary syndrome. U-shaped relationships were observed for the incidence rates of major adverse cardiac or cerebrovascular events (MACCE, a composite endpoint of cardiovascular death, myocardial infarction, and stroke), revascularization, and hospitalization for heart failure (HF). After adjusting for confounding factors, higher PP at discharge was associated with an increased risk of MACCE (adjusted hazard ratio 1.41; 95 %CI, 1.06–1.87 in Q5 [73.9 ± 9.3 mmHg]). Evaluating PP change revealed a U-shaped association with MACCE (1.50; 1.11–2.02 in Q1 and 1.47; 0.98–2.20 in Q5). Additionally, Q5 had a higher risk for hospitalization for HF (1.37; 1.00–1.88).

**Conclusions:**

Our findings demonstrate a U-shaped association between changes in PP and cardiovascular outcomes. This data suggests the significance of blood pressure control during hospitalization for patients who have undergone PCI.

## 1 Introduction

Blood pressure (BP) is a major modifiable risk factor for coronary artery disease (CAD) [1], [2]. Lowering BP in patients with hypertension reduces their risk of developing CAD [3], [4]. For patients with established CAD or heart failure (HF), previous studies have reported a J-shaped phenomenon between systolic BP (SBP) and diastolic BP (DBP) and cardiovascular outcomes, indicating that both higher and lower BP levels elevate the risk of poor prognosis [5], [6], [7], [8]. The relationship between SBP and DBP is better represented by pulse pressure (PP) [9]. PP reflects increased large artery stiffness and is an independent risk factor for new-onset CAD [9], [10], [11], [12], [13]. Additionally, in established CAD, PP serves as a prognostic risk factor for cardiovascular outcomes. [14], [15] However, prior studies have primarily focused on the prognostic implications of PP at baseline enrollment [15]. Limited data exist regarding the predictive value of changes in PP during hospitalization for patients with CAD who have undergone percutaneous coronary intervention (PCI).

This study aimed to evaluate the association between changes in PP and cardiovascular outcomes in patients with CAD who underwent PCI using the Clinical Deep Data Accumulation System (CLIDAS) database [16]. We also assessed the association of baseline PP with the outcomes to confirm the previous findings demonstrating the J- or U-shaped phenomenon in this patient population.

## 2 Methods

The CLIDAS database is a multimodal system that directly acquires clinical data from hospital information systems (HIS). Implemented in six university hospitals and the national cardiovascular center in Japan, CLIDAS was developed as part of the Japan Ischemic Heart Disease Multimodal Prospective Data Acquisition for Precision Treatment project launched in 2015 [16]. This project aimed to establish a standardized electronic system based on HIS to capture medical records and other clinical data for clinical studies. Briefly, data from HIS, picture archiving and communication systems, and physiology servers are linked to a multi-purpose clinical data repository system (MCDRS) through the Standardized Structured Medical Record Information eXchange2 (SS-MIX2) standard and extended storage. Data managers and researchers at each facility collect patients’ background information and follow-up data. After anonymization, the facilities send their data to the CLIDAS server through their individual MCDRS servers. CLIDAS collects data following the SS-MIX/SS-MIX2 standard format developed by Japan’s Ministry of Health, Labour and Welfare. Finally, researchers analyze the data stored on the CLIDAS server.

BP was measured according to usual practices at each facility at hospitalization before PCI and at discharge; these measurements were not standardized. The change in PP (ΔPP) was calculated by subtracting the BP at discharge from the BP at hospitalization (PP at hospitalization − PP at discharge).

We identified 9,690 patients who underwent PCI in the CLIDAS database between April 2013 and March 2019. Due to missing BP data, 982 patients were excluded (Supplementary Table 1). This resulted in a final analysis population of 8,708 patients with CAD.

The study protocol adhered to the ethical guidelines in the Declaration of Helsinki (1975) and was approved by the institutional ethics committees of all participating facilities. Since the CLIDAS data was anonymized, the requirement for informed consent was waived.

A major adverse cardiac or cerebrovascular event (MACCE) was defined as the first occurrence of any of the following: cardiovascular death, nonfatal stroke, or nonfatal myocardial infarction (MI). Data regarding these events were collected from medical records at each facility.

### 2.1 Statistical methods

Clinical characteristics were described by quintiles of ΔPP. Summary statistics were presented as mean standard deviation, median (interquartile range), and numbers (percentages), as appropriate. One-way analysis of variance was used to compare continuous normally distributed variables across the five groups. The Kruskal-Wallis test was employed for continuous non-normally distributed variables, and the Fisher exact test was used for categorical variables.

We estimated incidence rates using Poisson regression models with ΔPP modeled via restricted cubic spline curves. Multivariable Cox regression models were used to assess the association between changes in PP and the occurrence of MACCE, revascularization, or hospitalization for HF. Age, sex, and baseline PP at discharge were focused on models as covariates. Backward selection was then used to select additional variables from the following list: diabetes, dyslipidemia, smoking, atrial fibrillation, body mass index, estimated glomerular filtration rate (eGFR), number of coronary artery stenoses, brain natriuretic peptide (BNP), left ventricular ejection fraction (LVEF), history of MI, history of coronary artery bypass graft, history of PCI, and history of hospitalization for HF. We additionally assessed the association between the PP at discharge and patient outcomes.

Since changes in PP may differ based on the presence of acute coronary syndrome (ACS) or chronic coronary syndrome (CCS), we conducted a subgroup analysis to explore potential differences in these groups. Statistical significance was set at *p* < 0.05. All analyses were performed STATA 17 (College Station, TX).

## 3 Results

The study included 8,708 participants with a mean age of 70 ± 11 years, with 22 % being women (Table 1). SBP at hospitalization and discharge were 130 ± 23 mmHg and 119 ± 16 mmHg, respectively. Similarly, DBP and PP also showed a decrease from hospitalization to discharge (Table 1). The changes in PP across the quintiles were: −18.0 ± 9.9 in Q1 (n = 1755), −3.8 ± 2.6 in Q2 (n = 1873), 3.7 ± 2.0 in Q3 (n = 1671), 11.3 ± 2.6 in Q4 (n = 1671), and 27.5 ± 11.2 mmHg in Q5 (n = 1738). Interestingly, there was no significant trend in baseline characteristics across the quintiles, except for the highest prevalence of ACS observed in Q5 (Table 1).

**Table 1:** Baseline characteristics by the change of pulse pressure in the overall cohort.

	Overall (n = 8708)	Q1 (n = 1755)	Q2 (n = 1873)	Q3 (n = 1671)	Q4 (n = 1671)	Q5 (n = 1738)	*p* value
ΔPP (hospitalization-discharge), mmHg	3.9 ± 16.7	−18.0 ± 9.9	−3.8 ± 2.6	3.7 ± 2.0	11.3 ± 2.6	27.5 ± 11.2	
Age, years	70 ± 11	71 ± 11	69 ± 11	69 ± 11	70 ± 11	71 ± 11	<0.001
Women, n (%)	1924 (22 %)	384 (22 %)	361 (19 %)	356 (21 %)	374 (22 %)	449 (26 %)	<0.001
Body mass index, kg/m^2^	24.1 ± 3.8	24.0 ± 4.0	24.3 ± 3.8	24.2 ± 3.6	24.2 ± 4.0	24.1 ± 3.6	0.11
Systolic BP at hospitalization, mmHg	130 ± 23	115 ± 19	122 ± 17	127 ± 17	134 ± 18	151 ± 23	
Systolic BP at discharge, mmHg,	119 ± 16	127 ± 18	120 ± 16	118 ± 15	117 ± 15	115 ± 16	
Diastolic BP at hospitalization, mmHg	72 ± 15	72 ± 16	71 ± 14	71 ± 14	72 ± 15	74 ± 17	
Diastolic BP at discharge, mmHg	66 ± 10	65 ± 10	66 ± 10	65 ± 10	66 ± 10	66 ± 11	
Heart rate at hospitalization, bpm	74 ± 17	75 ± 18	73 ± 16	73 ± 16	73 ± 16	75 ± 19	<0.001
Heart rate at discharge, bpm	68 ± 11	68 ± 12	68 ± 11	68 ± 10	68 ± 11	69 ± 11	0.67
Current or ever smoker, n (%)	1864 (39 %)	365 (37 %)	430 (42 %)	364 (40 %)	335 (38 %)	370 (37 %)	0.09
Dyslipidemia, n (%)	6906 (80 %)	1364 (78 %)	1478 (79 %)	1314 (79 %)	1381 (83 %)	1369 (79 %)	0.009
Hypertension, n (%)	7180 (83 %)	1457 (84 %)	1516 (81 %)	1363 (82 %)	1384 (83 %)	1460 (84 %)	0.1
Diabetes, n (%)	3680 (43 %)	750 (43 %)	796 (43 %)	685 (41 %)	701 (42 %)	748 (43 %)	0.75
Dyslipidemia, n (%)	6906 (80 %)	1364 (78 %)	1478 (79 %)	1314 (79 %)	1381 (83 %)	1369 (79 %)	0.009
Peripheral artery disease, n (%)	600 (8 %)	140 (9 %)	111 (7 %)	110 (7 %)	110 (7 %)	129 (8 %)	0.12
Atrial fibrillation, n (%)	453 (5 %)	101 (6 %)	99 (5 %)	77 (5 %)	88 (5 %)	88 (5 %)	0.66
Previous hospitalization for HF, n (%)	577 (7 %)	134 (8 %)	126 (7 %)	102 (6 %)	109 (7 %)	106 (6 %)	0.33
Previous PCI, n (%)	1835 (21 %)	379 (22 %)	386 (21 %)	371 (22 %)	367 (22 %)	332 (19 %)	0.15
Previous CABG, n (%)	481 (6 %)	105 (6 %)	97 (5 %)	98 (6 %)	87 (5 %)	94 (5 %)	0.75
Previous MI, n (%)	1364 (16 %)	286 (16 %)	312 (17 %)	261 (16 %)	275 (17 %)	230 (13 %)	0.029
Previous history of stroke, n (%)	931 (11 %)	201 (12 %)	199 (11 %)	163 (10 %)	156 (9 %)	212 (12 %)	0.042
Acute coronary syndrome, n (%)	3721 (43 %)	718 (41 %)	699 (37 %)	643 (38 %)	699 (42 %)	962 (55 %)	<0.001

*Angiographic Findings, n (%)*							
LAD (including LMT)	6475 (81 %)	1317 (82 %)	1398 (82 %)	1223 (80 %)	1231 (80 %)	1306 (82 %)	0.42
RCA	4924 (62 %)	1037 (64 %)	1027 (60 %)	951 (62 %)	935 (61 %)	974 (61 %)	0.11
LCX	4353 (55 %)	923 (57 %)	895 (52 %)	827 (54 %)	812 (53 %)	896 (57 %)	0.014
Multiple vessel disease	3997 (50 %)	844 (52 %)	827 (48 %)	774 (51 %)	740 (48 %)	812 (51 %)	0.1
Triglyceride (median), mg/dL	117[86, 165]	115 [85, 158]	115 [86, 166]	120 [88, 171]	122 [86, 169]	115 [85, 163]	0.022
HDL cholesterol, mg/dL	47 ± 13	46 ± 13	47 ± 13	47 ± 13	47 ± 13	46.1 ± 13.1	0.2
LDL cholesterol, mg/dL	96 ± 31	95 ± 30	95 ± 31	96 ± 31	97 ± 31	99.7 ± 31.2	<0.001
LVEF, %	57 ± 14	57 ± 14	57 ± 14	58 ± 14	58 ± 14	57.5 ± 13.4	0.035
eGFR, mL/min/1.73 m^2^	59 ± 24	55 ± 25	60 ± 23	60 ± 23	61 ± 24	59.3 ± 26.5	<0.001
BNP (median), pg/mL	75 [29, 220]	93 [33, 273]	64 [25, 184]	63 [28, 193]	69 [27, 197]	85 [33, 233]	<0.001
Beta blockers, n (%)	4927 (57 %)	975 (56 %)	1032 (55 %)	924 (55 %)	940 (56 %)	1056 (60.8 %)	0.003
Statins, n (%)	4671 (54 %)	932 (53 %)	1071 (57 %)	933 (56 %)	935 (56 %)	800 (46.0 %)	<0.001

Abbreviations: BP, blood pressure; BNP, B-type natriuretic peptide; CABG, coronary artery bypass grafting; eGFR, estimated glomerular filtration rate; HDL, high-density lipoprotein; HF, heart failure; LAD, left anterior descending artery; LCX, left circumflex artery; LDL, low-density lipoprotein; LMT, left main trunk; LVEF, left ventricular ejection fraction; MI, myocardial infarction; PCI, percutaneous coronary intervention; PP, pulse pressure; RCA, right coronary artery.

During a median follow-up period of 944 days, 702 MACCE were observed.

Event rates (per 1,000 person-years) for MACCE showed a U-shaped association with changes in PP. Individuals with Q1 (lowest decrease, 5.7 [95 % confidence interval [95 %CI] 4.7–7.0)] and Q5 (highest increase, 8.2 [7.0–9.7]) had higher rates compared to the reference group (6.9 [5.8–8.1]) (Supplemental Table 2).

**Fig. 1:**
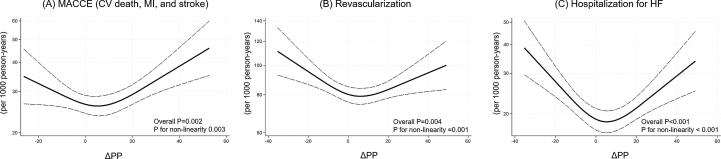
Incidence rates per 1,000 person-years of (A) major adverse cardiac or cerebrovascular event [MACCE; defined as a composite of cardiovascular (CV) death, myocardial infarction (MI), and stroke], (B) revascularization, and (C) hospitalization for heart failure (HF) across changes in pulse pressure (ΔPP). ΔPP is calculated by subtracting blood pressure at discharge from the blood pressure at hospitalization. The solid line represents the incidence rate, and the dashed lines represent the 95% confidence intervals.

For revascularization, the rates were 21.1(18.9–23.6) in Q1, 25.4 (23.0–28.1) in Q2, 22.1 (20.0–24.5) in Q3, 23.6 (21.2–26.1) in Q4, and 23.3 (20.1–25.9) in Q5. For hospitalization for HF, the rates were 5.1 (4.1–6.3) in Q1, 6.4 (5.3–7.8) in Q2, 6.1 (5.1–7.3) in Q3, 5.3 (4.3–6.5) in Q4, and 6.7 (5.6–8.0) in Q5 (Supplemental Table 2). This suggests that a U-shaped association may be observed between PP change and outcomes (Fig. 1).

After adjusting for age, sex, baseline PP, eGFR, number of coronary artery stenosis, BNP, and LVEF, the analysis confirmed the U-shaped relationship between PP change and MACCE. Compared to the reference group (Q3), a decrease in PP (Q1) was associated with a 50 % increased risk of MACCE (adjusted hazard ratio [HR] 1.50, 95 %CI 1.11–2.02, *p* = 0.008) (Table 2). Conversely, an increase in PP (Q4 and Q5) was also associated with an increased risk of MACCE (HR 1.67, 1.24–2.26, *p* = 0.001 in Q4 and HR 1.33, 0.98–1.82, *p* = 0.07 in Q5). Additionally, higher PP was linked to a higher risk of hospitalization for HF (1.37, 1.00–1.85, *p* = 0.05) (Table 2). Similar findings were observed when analyzing the association between baseline PP and the outcomes (Supplemental Table 3). Notably, the U-shaped association between PP change and outcomes remained consistent when stratified by ACS or CCS (Table 2).

**Table 2:** Association of pulse pressure change with major adverse cardiac or cerebrovascular events, revascularization, and heart failure due to hospitalization.

ΔPP (hospitalization-discharge)	MACCE		Revascularization		HF hospitalization	
	HR (95 %CI)	*p* value	HR (95 %CI)	*p* value	HR (95 %CI)	*p* value
Q1 (−18.0 ± 9.9 mmHg)						
Overall (n = 1755)	1.50 (1.11–2.02)	0.008	0.98 (0.82–1.18)	0.84	0.88 (0.63–1.23)	0.45
ACS (n = 718)	1.58 (1.01–2.47)	0.045	0.90 (0.66–1.21)	0.48	0.70 (0.41–1.19)	0.19
CCS (n = 1037)	1.47 (0.98–2.20)	0.06	1.03 (0.81–1.30)	0.81	1.06 (0.69–1.65)	0.78

Q2 (−3.8 ± 2.6 mmHg)						
Overall (n = 1873)	1.22 (0.90–1.67)	0.21	0.99 (0.82–1.18)	0.87	1.34 (0.98–1.83)	0.07
ACS (n = 699)	1.47 (0.93–2.34)	0.10	1.11 (0.84–1.49)	0.46	1.29 (0.79–2.09)	0.30
CCS (n = 1174)	1.09 (0.72–1.66)	0.68	0.91 (0.73–1.15)	0.44	1.39 (0.92–2.10)	0.12

Q3 (3.7 ± 2.0 mmHg)						
Overall (n = 1671)	Reference		Reference		Reference	
ACS (n = 643)	Reference		Reference		Reference	
CCS (n = 1028)	Reference		Reference		Reference	

Q4 (11.3 ± 2.6 mmHg)						
Overall (n = 1671)	1.67 (1.24–2.26)	0.001	1.21 (1.01–1.44)	0.036	1.26 (0.90–1.74)	0.17
ACS (n = 699)	1.76 (1.13–2.75)	0.01	1.24 (0.94–1.64)	0.13	1.08 (0.65–1.79)	0.76
CCS (n = 972)	1.59 (1.06–2.39)	0.03	1.19 (0.94–1.49)	0.15	1.37 (0.89–2.45)	0.16

Q5 (27.5 ± 11.2 mmHg)						
Overall (n = 1738)	1.33 (0.98–1.82)	0.07	1.13 (0.94–1.35)	0.20	1.37 (1.00–1.88)	0.05
ACS (n = 962)	1.26 (0.80–1.96)	0.32	1.15 (0.88–1.51)	0.30	1.16 (0.73–1.85)	0.52
CCS (n = 776)	1.40 (0.91–2.16)	0.13	1.11 (0.86–1.43)	0.42	1.58 (1.03–2.45)	0.04

Abbreviations: ACS, acute coronary syndrome; CCS, chronic coronary syndrome; HF, heart failure; MACCE, major adverse cardiac or cerebrovascular event; PP, pulse pressure.

The final model was adjusted for age, sex, baseline PP, estimated glomerular filtration rate, number of coronary artery stenosis, brain natriuretic peptide, and left ventricular ejection fraction.

## 4 Discussion

Our study, one of the largest to quantify the prognostic value of changes in PP during hospitalization, offers valuable insights into the predictors of PP and their association with MACCE and HF hospitalization in patients with CAD who underwent PCI. The key novel finding is that changes in PP during hospitalization emerged as a prognostic risk factor for both MACCE and HF hospitalization. This suggests a potentially under-recognized but important role for PP changes in patient prognosis, and the potential utility of PP for a more accurate risk assessment after PCI. We observed a U-shaped relationship between changes in PP and cardiovascular outcomes, along with confirming the established link between baseline PP and outcomes.

Prior research on PP has primarily focused on its prognostic value for developing CAD in hypertensive patients [9], [10], [11], [12], [17]. This is because a larger PP reflects increased large artery stiffness, a factor contributing to atherosclerosis [18], [19]. Indeed, a previous study demonstrated a positive correlation between PP and coronary calcium volume, mass, density, and Agatston score in the general population [20]. Additionally, the Framingham Heart Study showed that in middle-aged and elderly individuals (50–79 years), CAD risk increased with lower DBP at any SBP level ≥120 mmHg, suggesting the importance of higher PP for CAD risk [10]. The study also reported that neither SBP nor DBP was superior to PP in predicting risk, implying PP’s usefulness for risk stratification [10]. Existing research on established CAD patients aligns with our findings, demonstrating that baseline PP is a prognostic risk factor for cardiovascular outcomes.

Our findings extend the concept of visit-to-visit BP variability from the STABILITY trial sub-analysis, which showed that higher variability in both SBP and DBP independently predicts increased cardiovascular risk in CAD patients, regardless of mean BP [21]. Our study suggests that both increased and decreased PP are associated with poor cardiovascular outcomes in patients undergoing PCI. This highlights the importance clinicians to consider not only SBP and DBP but also PP when monitoring these patients. The ability to predict patient outcomes based on PP changes from admission to discharge can inform more personalized and effective management strategies. By incorporating PP into routine assessments, clinicians may improve the detection and management of cardiovascular conditions, ultimately leading to better patient outcomes.

As a retrospective observational study, our research cannot establish causality. However, it can generate hypotheses for future clinical trials and help identify high-risk patients who might benefit from stricter BP control after PCI.

This study has several limitations. First, missing BP data in 982 (10.1 %) of the 9,690 CLIDAS participants between April 2013 and March 2019 could introduce selection bias. However, minimal differences in clinical characteristics between included and excluded patients suggest minimal bias (Supplemental Table 1). Second, we lack information on valvular diseases (e.g. aortic regurgitation) and BP treatment during hospitalization, which could be missing confounders when assessing the prognostic value of PP. Finally, with the CLIDAS sample consisting almost entirely of Japanese individuals, the results may not be generalizable to other ethnic and socioeconomic groups.

## 5 Conclusions

This large, hospital-based study suggests that both increases and decreases in PP during hospitalization are associated with a higher risk of MACCE or hospitalization for HF in patients with CAD who underwent PCI. These results suggest the need for caution when managing BP during hospitalization for this patient population.

## CRediT authorship contribution statement

**Kotaro Nochioka:** Writing – original draft, Visualization, Software, Methodology, Investigation, Formal analysis, Data curation, Conceptualization. **Masaharu Nakayama:** Writing – review & editing, Supervision, Data curation, Conceptualization. **Naoyuki Akashi:** Writing – review & editing, Methodology, Data curation. **Tetsuya Matoba:** Writing – review & editing, Resources, Project administration, Investigation, Data curation. **Takahide Kohro:** Writing – review & editing, Resources, Project administration, Methodology, Data curation. **Yusuke Oba:** Writing – review & editing, Data curation. **Tomoyuki Kabutoya:** Writing – review & editing, Resources, Data curation. **Yasushi Imai:** Writing – review & editing, Data curation. **Kazuomi Kario:** Writing – review & editing, Supervision. **Arihiro Kiyosue:** Writing – review & editing, Data curation. **Yoshiko Mizuno:** Writing – review & editing, Data curation. **Takamasa Iwai:** Writing – review & editing, Data curation. **Yoshihiro Miyamoto:** Writing – review & editing, Supervision, Data curation. **Masanobu Ishii:** Writing – review & editing, Data curation. **Taishi Nakamura:** Writing – review & editing, Data curation. **Kenichi Tsujita:** Writing – review & editing, Supervision. **Hisahiko Sato:** Writing – review & editing, Supervision, Project administration, Data curation. **Hideo Fujita:** Writing – review & editing, Supervision, Project administration, Data curation, Conceptualization. **Ryozo Nagai:** Writing – review & editing, Supervision, Funding acquisition, Conceptualization.

## Declaration of competing interest

The authors declare the following financial interests/personal relationships which may be considered as potential competing interests: **TK** has received scholarship funding from Abbott. **KK** has received **a** research grant from Otsuka Pharmaceutical, Sanwa Kagaku Kenkyusho, Daiichi Sankyo, MSD, Astellas Pharma, Eisai, Taisho Pharmaceutical, Sumitomo Dainippon Pharma, Takeda Pharmaceutical, Mitsubishi Tanabe Pharma, Teijin Pharma, Boehringer Ingelheim Japan, Bristol-Myers Squibb, Mochida Pharmaceutical; Consulting fees from Kyowa Kirin, Sanwa Kagaku Kenkyusho, Mochida Pharmaceutical; Honoraria from Otsuka Pharmaceuticals, Daiichi Sankyo, Novartis Pharma, Mylan EPD; Participation in Advisory Board of Daiichi Sankyo, Novartis Pharma. **HF** received scholarship funds from Abbott Vascular, speaking honoraria from Novartis and Otsuka Pharmaceutical Co., Ltd., and served as a consultant for Mehergen Group Holdings, Inc. **KT** has received personal fees from Abbott Medical Co., Ltd., personal fees from Amgen K.K., personal fees from AstraZeneca K.K., personal fees from Bayer Yakuhin, Ltd., personal fees from Daiichi Sankyo Co., Ltd., personal fees from Medtoronic Japan Co., Ltd., personal fees from Kowa Pharmaceutical Co. Ltd., personal fees from Novartis Pharma K.K., personal fees from Otsuka Pharmaceutical Co.,Ltd., personal fees from Pfizer Japan Inc., personal fees from Janssen Pharmaceutical K.K., grants from PPD-Shin Nippon Biomedical Laboratories K.K., grants from Alexion Pharmaceuticals, Inc., grants from Abbott Medical Co., Ltd., grants from Bayer Yakuhin, Ltd., grants from Boehringer Ingelheim Japan, grants from Daiichi Sankyo Co., Ltd., grants from ITI Co.,Ltd., grants from ONO PHARMACEUTICAL CO., LTD., grants from Otsuka Pharmaceutical Co.,Ltd., grants from Takeda Pharmaceutical Co., Ltd., other from Abbott Japan Co., Ltd., other from Boston Scientific Japan K.K., other from Fides-one, Inc., other from GM Medical Co., Ltd., other from ITI Co.,Ltd., other from Kaneka Medix Co., Ltd., other from NIPRO CORPORATION, other from TERUMO Co, Ltd., other from Abbott Medical Co., Ltd., other from Boston Scientific Japan K.K., other from Cardinal Health Japan, other from Fukuda Denshi Co., Ltd., other from Japan Lifeline Co.,Ltd., other from Medical Appliance Co., Ltd., other from Medtronic Japan Co., Ltd., outside the submitted work. **TM** has received lecture fees (Abbott, Bayer Yakuhin, and MSD) and research funding (Amgen, Bayer Yakuhin, and Kowa). **YM** received research funds from Kowa Company, Ltd., within the submitted work and from Tokyo Marine and Nichido Fire Insurance Co., Ltd., Fujitsu Co., Ltd., Softbank Co., Ltd., Saraya Co., Ltd., and Meiji Yasuda Life Insurance Company outside of the submitted work.
