# Supplementary material for: Change in pulse pressure and cardiovascular outcomes after percutaneous coronary intervention: The CLIDAS study
Source: Int J Cardiol Heart Vasc. 2024 May 24;53:101430. doi: 10.1016/j.ijcha.2024.101430 (PMC11368593; doi:10.1016/j.ijcha.2024.101430)
Supplement: Supplementary Data 1 [file mmc1.docx]

# Supplemental materials

**Title: Change in Pulse Pressure and Cardiovascular Outcomes After Percutaneous Coronary Intervention: The CLIDAS Study**

**Supplemental Table 1.** Baseline characteristics by missing blood pressure

|  | **Blood pressure**  **Available**  (n=8708) | **Blood pressure**  **not available**  (n=982) | **P value** |
| --- | --- | --- | --- |
| Age | 70±11 | 70± 11 | 0.53 |
| Women | 1924 (22%) | 259 (26%) | 0.002 |
| Body mass index | 24.1 ± 3.8 | 23.9 ± 3.9 | 0.08 |
| Hypertension | 7180 (83%) | 717 (74%) | <0.001 |
| Diabetes | 3680 (43%) | 481 (50%) | <0.001 |
| Dyslipidemia | 6906 (80%) | 628 (65%) | <0.001 |
| Current or ever smoker | 1864 (39%) | 194 (34%) | <0.001 |
| Peripheral artery disease | 600 (8%) | 139 (15%) | <0.001 |
| Atrial fibrillation | 453 (5%) | 40 (4%) | 0.14 |
| Previous hospitalization for HF | 577 (7%) | 60 (6%) | 0.54 |
| Previous PCI | 1835 (21%) | 160 (16%) | <0.001 |
| Previous CABG | 481 (6%) | 36 (4%) | 0.014 |
| Previous MI | 1364 (16%) | 115 (12%) | 0.001 |
| Previous history of Stroke | 931 (11%) | 112 (12%) | 0.47 |
| Acute coronary syndrome | 4987 (57%) | 568 (58%) | 0.73 |
| Angiographic Findings |  |  |  |
| LM LAD | 6475 (81%) | 548 (58%) | <0.001 |
| RCA | 4924 (62%) | 451 (48%) | <0.001 |
| LCX | 4353 (55%) | 385 (41%) | <0.001 |
| Multi VD | 3997 (50%) | 356 (38%) | <0.001 |
| left ventricular ejection fraction | 57.3 ± 13.8 | 57.6 ± 11.6 | 0.58 |
| eGFR | 58.7 ± 24.2 | 54.9 ± 24.6 | <0.001 |
| Median BNP | 75 [29, 220] | 72 [27, 224] | 0.82 |
| Beta blockers | 4927 (57%) | 598 (61%) | 0.01 |
| Statins | 4671 (54%) | 646 (66%) | <0.001 |

Abbreviations: BP, blood pressure; BNP, B-type natriuretic peptide; CABG, coronary artery bypass grafting; eGFR, estimated glomerular filtration rate; HF, heart failure, LAD, left anterior descending artery; LCX, left circumflex artery; LMT, left main trunk; MI, myocardial infraction; RCA, right coronary artery.

**Supplemental Table 2. Incidence rates for the outcomes by quintiles of ΔPP.**

|  | **MACCE**  **[/1000-years (95%CI)]** | **Revascularization**  **[/1000-years (95%CI)]** | **Hospitalization for HF [/1000-years (95%CI)]** |
| --- | --- | --- | --- |
| **Q1** | 5.7 (4.7-7.0) | 21.1(18.9-23.6) | 5.1 (4.1-6.3) |
| **Q2** | 10.3 (8.9-11.9) | 25.4 (23.0-28.1) | 6.4 (5.3-7.8) |
| **Q3 (reference)** | 6.9 (5.8-8.1) | 22.1 (20.0-24.5) | 6.1 (5.1-7.3) |
| **Q4** | 8.3 (7.0-9.8) | 23.6 (21.2-26.1) | 5.3 (4.3-6.5) |
| **Q5** | 8.2 (7.0-9.7) | 23.3 (20.1-25.9) | 6.7 (5.6-8.0) |

**Abbreviation: HF, heart failure; MACCE, major adverse cardiac or cerebrovascular event; PP, pulse pressure.**

**Supplemental Table 3.** Association of PP at baseline with MACCE, revascularization, and HF hospitalization.

|  | **MACCE** | | **Revascularization** | | **HF hospitalization** | |
| --- | --- | --- | --- | --- | --- | --- |
| Baseline PP | HR (95%CI) | P value | HR (95%CI) | P value | HR (95%CI) | P value |
| Q1(36.4 ± 5.7mmHg) | 1.22 (0.90-1.67) | 0.20 | 0.81 (0.67-0.98) | 0.03 | 1.24 (0.90-1.70) | 0.19 |
| Q2(46.1 ± 2.0mmHg) | 1.32 (0.98-1.78) | 0.07 | 1.00 (0.84-120) | 0.96 | 1.09 (0.78-1.51) | 0.63 |
| Q3（52.5 ± 1.8mmHg） | Reference | | Reference | | Reference | |
| Q4(59.7 ± 2.4mmHg) | 1.21 (0.90-1/62) | 0.21 | 1.00 (0.84-1.20) | 0.96 | 1.12 (0.81-1.55) | 0.49 |
| Q5(73.9 ± 9.3mmHg) | 1.41 (1.06-1.87) | 0.02 | 1.21 (1.02-1.45) | 0.03 | 1.22 (0.89-1.67) | 0.21 |

**Abbreviation: HF, heart failure; MACCE, major adverse cardiac or cerebrovascular event; PP, pulse pressure.**

**The final model was adjusted for age, sex, estimated glomerular filtration rate, number of coronary artery stenosis, brain natriuretic peptide, and left ventricular ejection fraction.**

**Supplemental Figure 1. Association of PP at baseline with MACCE, revascularization, and HF hospitalization.**

**Incidence rates of the major adverse cardiac or cerebrovascular event [****MACCE; a composite of cardiovascular (CV) death, myocardial infarction (MI), and stroke] (A), revascularization (B), or hospitalization for heart failure (HF) (C) across baseline pulse pressure (PP). Solid line indicates incidence rate per 1000 person-years. Dashed lines indicate the 95% confidence intervals for these incidence rates.**
